# LGWheatNet: A Lightweight Wheat Spike Detection Model Based on Multi-Scale Information Fusion

**DOI:** 10.3390/plants14071098

**Published:** 2025-04-02

**Authors:** Zhaomei Qiu, Fei Wang, Tingting Li, Chongjun Liu, Xin Jin, Shunhao Qing, Yi Shi, Yuntao Wu, Congbin Liu

**Affiliations:** 1 College of Agricultural Equipment Engineering, Henan University of Science and Technology, Luoyang 471000, China; 2 Science & Technology Innovation Center for Completed Set Equipment, Longmen Laboratory, Luoyang 471003, China; 3 Hebei Nonghaha Agricultural Machinery Group Co., Ltd., Shijiazhuang 052560, China

**Keywords:** computer vision, count, deep learning, LightDetect, feature fusion, slicing aided hyper inference

## Abstract

Wheat spike detection holds significant importance for agricultural production as it enhances the efficiency of crop management and the precision of operations. This study aims to improve the accuracy and efficiency of wheat spike detection, enabling efficient crop monitoring under resource-constrained conditions. To this end, a wheat spike dataset encompassing multiple growth stages was constructed, leveraging the advantages of MobileNet and ShuffleNet to design a novel network module, SeCUIB. Building on this foundation, a new wheat spike detection network, LGWheatNet, was proposed by integrating a lightweight downsampling module (DWDown), spatial pyramid pooling (SPPF), and a lightweight detection head (LightDetect). The experimental results demonstrate that LGWheatNet excels in key performance metrics, including Precision, Recall, and Mean Average Precision (mAP50 and mAP50-95). Specifically, the model achieved a Precision of 0.956, a Recall of 0.921, an mAP50 of 0.967, and an mAP50-95 of 0.747, surpassing several YOLO models as well as EfficientDet and RetinaNet. Furthermore, LGWheatNet demonstrated superior resource efficiency with a parameter count of only 1,698,529 and GFLOPs of 5.0, significantly lower than those of competing models. Additionally, when combined with the Slicing Aided Hyper Inference strategy, LGWheatNet further improved the detection accuracy of wheat spikes, especially for small-scale targets and edge regions, when processing large-scale high-resolution images. This strategy significantly enhanced both inference efficiency and accuracy, making it particularly suitable for image analysis from drone-captured data. In wheat spike counting experiments, LGWheatNet also delivered exceptional performance, particularly in predictions during the filling and maturity stages, outperforming other models by a substantial margin. This study not only provides an efficient and reliable solution for wheat spike detection but also introduces innovative methods for lightweight object detection tasks in resource-constrained environments.

## 1. Introduction

In modern agricultural practices, wheat stands as one of the world’s most widely cultivated staple crops. Accurate counting of wheat spikes plays a critical role in yield estimation and field management guidance [1,2,3]. Wheat spike counting is a vital component in both agricultural production and research, as it not only impacts the accurate forecasting of wheat yields but also influences the enhancement of agricultural economic benefits and the optimization of resource allocation [4,5,6]. However, traditional wheat spike counting has primarily relied on manual field operations, a method that is not only inefficient but also prone to accuracy issues due to human factors [7]. With the expansion and modernization of agricultural production, these conventional counting methods can no longer meet the demands of large-scale, high-efficiency production in modern agriculture [8]. Currently, there is an urgent need in the agricultural sector to develop an efficient and accurate wheat spike counting method that can improve yield prediction accuracy and significantly enhance agricultural economic benefits.

With advancements in image processing technology, researchers have begun exploring automated wheat spike detection and counting by analyzing field images, significantly enhancing efficiency and accuracy [9]. Zhou et al. [10] developed a method using red–green–blue (RGB) images based on light intensity selection. By employing image segmentation, superpixel theory, and multi-feature optimization, along with the TWSVM-Seg model, they achieved rapid and accurate wheat spike counting. Fernandez-Gallego et al. [11] used thermal imaging technology to develop an automated image processing system, which leveraged contrast enhancement and filtering techniques to separate wheat spikes from other parts of the crop canopy based on temperature differences, enabling automatic wheat spike counting under field conditions. Hong et al. [12] introduced the CTHNet network, a hybrid attention network that integrates local features and global contextual information, designed for accurate wheat spike counting from RGB images. While image processing-based object detection methods offer advantages in real-time performance, interpretability, and reduced data annotation requirements, they exhibit notable limitations in terms of generalizability, feature robustness, detection precision, and adaptability. These constraints mean that when faced with complex real-world conditions, such methods typically underperform compared to deep learning approaches, limiting their applicability in broader scenarios [13,14].

With the rise of deep learning, especially the application of convolutional neural networks (CNNs), the technology for wheat spike detection and counting has advanced significantly. Deep learning models can automatically learn complex feature representations from images without the need for manually designed features, greatly enhancing detection accuracy and robustness [15,16,17]. Object detection models in deep learning are generally classified into two main categories: two-stage and single-stage models. Two-stage models typically consist of a Region Proposal Network (RPN) and a detection network. The RPN identifies potential target regions, or region proposals, which are then passed to the detection network for classification and recognition [18,19]. This approach yields precise region proposals, thus improving the accuracy of detection tasks [20,21]. Typical single-stage networks include the YOLO series [22], SSD [23], EfficientDet [24], and RetinaNet [25]. These models perform efficient and fast object detection by applying a sliding window across the entire image to predict bounding boxes and class confidence scores. In the object detection field, the YOLO series is particularly popular due to its rapid inference speed and high accuracy, offering swift image processing and excellent accuracy [26,27]. Furthermore, the YOLO models’ generalizability and flexible adjustment capabilities make them more adaptable and stable under varied field conditions [28]. Dandrifosse et al. [29] developed an automatic wheat spike detection and segmentation method based on deep learning using the YOLOv5 and DeepMAC models to count and segment wheat spikes from heading to maturity stages. Yang et al. [30] proposed a wheat spike detection and counting method combining YOLOv4 and CBAM, demonstrating strong robustness through training and testing on multiple datasets, which improved detection and counting accuracy in complex backgrounds. Xu et al. [31] introduced an automatic wheat spike counting model based on the minimal area intersection ratio algorithm and transfer learning. Using smartphone-collected images, along with K-means clustering and the YOLOv5 model, they achieved rapid wheat spike identification and counting. Gao Yunpeng [32] applied YOLOv3 and Mask R-CNN networks to the wheat spike detection task. The results indicated that Mask R-CNN achieved higher accuracy, while YOLOv3 demonstrated faster detection speed. Their recognition accuracies were 97.00% and 87.12%, respectively, with detection speeds per image of 0.94 s and 0.12 s. In the field of object detection, two-stage networks are widely used in applications where high accuracy is essential due to their precision. However, because two-stage models separately execute region proposal generation and target detection, they are computationally intensive, which imposes limitations on their use in real-time applications. Compared to two-stage networks, single-stage networks unify the region proposal and detection processes within a single architecture, directly predicting the class and location of targets in the input image. This approach enhances detection speed by eliminating the region proposal step.

Current wheat spike detection networks face two primary challenges. First, high-precision models often require handling extensive parameters and complex floating-point calculations, making deployment on resource-constrained devices difficult. Second, while lightweight models are more streamlined in terms of parameters and computational needs, their detection accuracy is often not optimal [33]. Additionally, the visual characteristics of wheat spikes change across different growth stages, demanding higher adaptability and generalization capabilities from the models [34]. This complexity requires models to be designed with a balance of efficiency and accuracy. The model needs to be sufficiently complex to capture subtle variations in wheat spike features, yet simple enough for effective deployment in resource-limited environments. Furthermore, it must be flexible enough to adapt to changes in wheat spike characteristics over time.

To address these challenges, this study introduces a series of innovative modules to construct a lightweight wheat spike detection network called LGWheatNet. The study selects a small-scale version of MobileNetv4 as the backbone architecture to ensure model lightweight and efficiency. By incorporating channel attention mechanisms, channel shuffling, and optimization of the activation function, the study improves MobileNetv4’s generic inverted residual bottleneck block (UIB) to develop a new SeCUIB module. Based on SeCUIB, the MobileNetv4 network is adjusted and combined with the SPPF module to construct a lightweight backbone network. Additionally, a lightweight downsampling module, DWDown, is created by combining pointwise convolution and depthwise convolution. The SeCUIB and DWDown modules are used to build the FPN + PAN neck network. Finally, a lightweight head network, LightDetect, is designed by integrating group convolutions and depthwise convolutions. Through the integration of these innovative modules, the LGWheatNet model achieves high detection accuracy while significantly reducing computational complexity, providing an effective solution for wheat spike detection in resource-constrained environments.

## 2. Materials and Methods

### 2.1. Study Location and Wheat Variety

**Figure 1:**
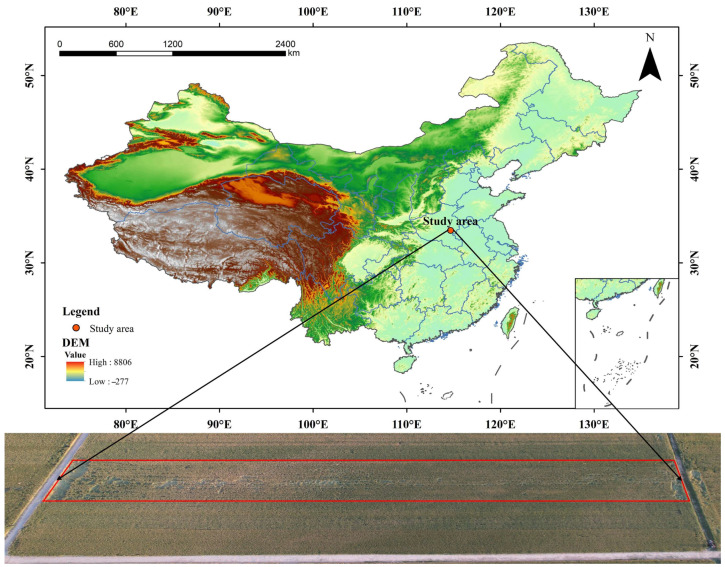
The geographical location of the study area.

The image data for this study were collected from the primary farmland in Yuanlao Township, Shangshui County, Zhoukou City, Henan Province. The site is geographically positioned at 114°41′3.73″ E longitude and 33°27′34.53″ N latitude, based on the CGCS2000 coordinate system provided by the Beidou navigation satellite system, as shown in Figure 1. Yuanlao Township has a flat terrain, sloping from higher elevations in the northwest to lower elevations in the southeast, and is characterized by a warm temperate continental monsoon climate. This climate is marked by moderate temperatures year-round and distinct seasonal changes, making it suitable for wheat cultivation. The wheat grown in this area is winter wheat, typically planted from October to November and harvested around June of the following year. The wheat variety used in this study was Shanhe 1028, which is well-suited to the southern part of the Huang-Huai winter wheat region in Henan Province, excluding the rice-stubble wheat areas south of the Huai River in Xinyang City and parts of southern Nanyang. It is primarily cultivated in irrigated plains across this region.

### 2.2. Dataset Construction

In this study, images of wheat spikes and grains in the field were collected using a Redmi K40 smartphone. The Redmi K40, released by Xiaomi, is equipped with a Qualcomm Snapdragon 870 processor, runs the MIUI 12 operating system based on Android 11, and features a 48-megapixel main rear camera, an 8-megapixel ultra-wide camera, and a 5-megapixel telephoto macro lens, along with a 20-megapixel front camera. The equipment was sourced from the manufacturer Foxconn, which is headquartered in Taipei, Taiwan. The image data includes wheat spike images from flowering, filling, and maturity stages of wheat grown in the large fields of Shangshui County, Zhoukou City, Henan Province, in June 2024. Data collection dates were 21 April, 13 May, and 27 May 2024, with the weather conditions (https://tianqi.2345.com/) listed in Table 1.

**Figure 2:**
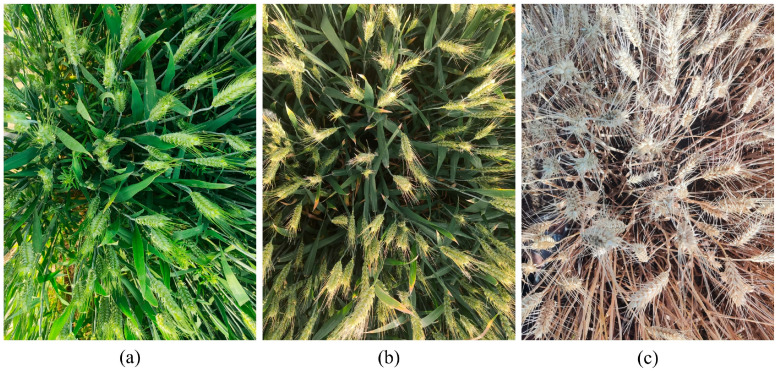
Representative images of wheat spikes at different growth stages in field conditions: (**a**) flowering wheat, (**b**) filling wheat, and (**c**) ripe wheat.

Using the Redmi K40, a total of 696 images of wheat spikes were collected, each with a resolution of 3000 × 4000 pixels and saved in .jpg format. To ensure clear image collection and the acquisition of multi-scale wheat spike information, we manually focused and captured wheat spike images at distances of 20 cm and 80 cm from the wheat spike, respectively. To maintain stable data capture and video stream detection, all images were taken from a top–down perspective. Additionally, to account for lighting variations that could affect detection results, images were captured under both cloudy and sunny conditions and at different times of the same day to ensure diversity in the wheat spike images. Sample images of raw field wheat spike captures are shown in Figure 2.

**Figure 3:**
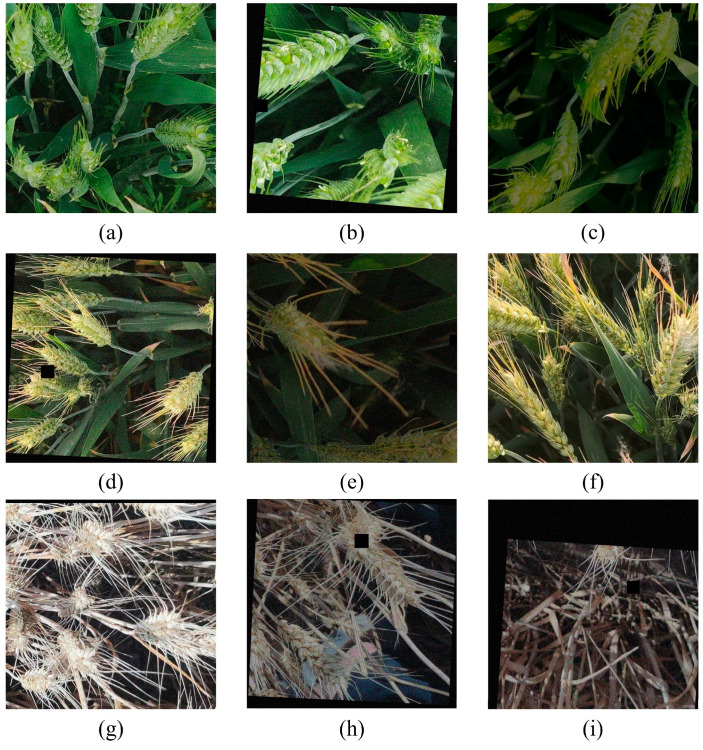
Sample wheat images after data augmentation: (**a**)—addition of random noise; (**b**)—random rotation, addition of random noise, and partial image occlusion; (**c**)—adjustment of image brightness; (**d**)—random rotation, partial image occlusion, and addition of random noise; (**e**)—adjustment of image brightness and addition of random noise; (**f**)—random mirroring; (**g**)—adjustment of image brightness; (**h**)—partial image occlusion, random rotation, and addition of random noise; (**i**)—random translation, partial image occlusion, addition of random noise, and random rotation.

The field images were cropped and processed to enhance model training efficiency and accuracy. First, the original images were resized from 3000 × 4000 pixels to 1000 × 1000 pixels to reduce data redundancy while retaining critical information. Data annotation was conducted using the labelImg software in a Python environment configured with Anaconda. The Python version used was 3.8, while the labelImg version was 1.8.6. The annotations were saved in the PASCAL visual object class (VOC) format as .xml files, with three designated categories: “flowerwheat”, “fillingwheat”, and “ripewheat”. The dataset was augmented using various image enhancement techniques to improve the model’s generalization and robustness. Specific augmentation methods included adding random noise, adjusting image brightness, random occlusion of parts of the images, image rotation, cropping, translation, and mirroring. Samples of the augmented images are displayed in Figure 3.

Finally, the dataset was randomly divided into three parts: training, validation, and test sets, with a ratio of 8:1:1. For the flowering stage, the test set contained 4380 samples, the training set 19,728 samples, and the validation set 3728 samples. In the filling stage, the test set contained 3570 samples, the training set 16,082 samples, and the validation set 3363 samples. For the maturity stage, the test set contained 3611 samples, the training set 17,254 samples, and the validation set 3730 samples.

### 2.3. Wheat Spike Detection Model Construction

#### 2.3.1. Construction of the Wheat Spike Detection Network’s Main Modules

In the current lightweight networks, the MobileNet series and ShuffleNet series have performed particularly well. The MobileNet series significantly reduces computational costs by using depthwise separable convolutions while maintaining high feature extraction capabilities, making it especially effective on resource-constrained devices. The module structure is simple and efficient, suitable for a wide range of mobile tasks [35,36]. The ShuffleNet series, on the other hand, optimizes the flow of information between feature maps by using channel shuffling and splitting techniques, improving computational resource utilization and further reducing computational and parameter costs [37,38].

**Figure 4:**
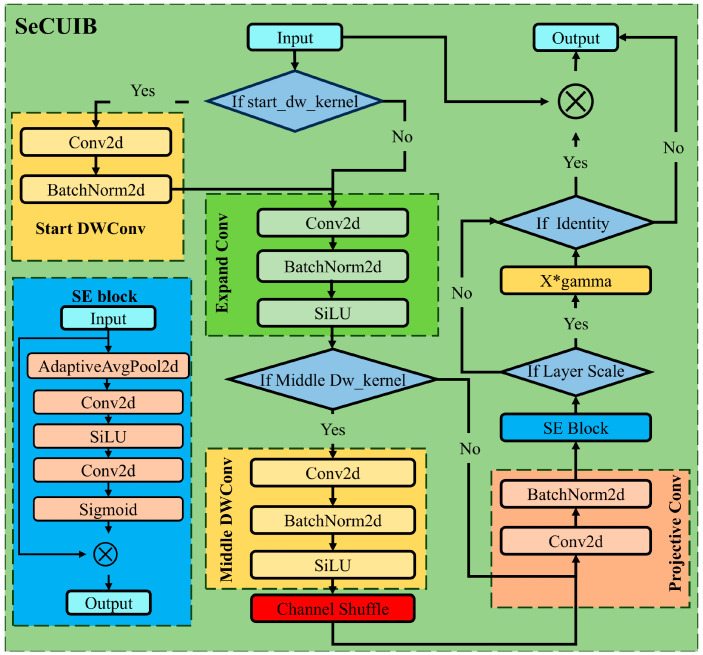
Structure of the SeCUIB module.

In this study, a new module was developed by introducing a channel attention mechanism, channel shuffling technique, and adjusting activation functions. This module, called the Squeeze-and-Excitation Channel Shuffle Universal Inverted Bottleneck (SeCUIB), is designed to improve feature extraction ability while maintaining computational efficiency. The SE attention mechanism adaptively recalibrates the feature responses of channels, allowing the model to focus on the feature channels that are most important for the wheat spike detection task [39,40]. This enhances the model’s ability to perceive wheat spike features while suppressing the interference of background noise. The channel shuffling technique, by reorganizing the channel dimension of the feature map, strengthens the inter-channel information flow, breaking the independence of channels and enabling the model to better capture the multi-scale features of wheat spikes [41,42]. The SiLU activation function further improves the model’s nonlinear expressive capability, enhancing its adaptability to complex scenes and enabling it to better handle challenges such as lighting variations and background noise [43]. The structure of the SeCUIB module is shown in Figure 4.

The main computational steps of the SeCUIB module can be divided into the following stages: initial depthwise convolution, expansion convolution, intermediate depthwise convolution, channel shuffling, projection convolution, SE attention mechanism, and residual connection.

The initial depthwise convolution stage is optional and is applied only when the kernel parameters of the initial depthwise convolution are not set to zero. The initial depthwise convolution performs preliminary feature recognition and extraction on the input feature map *x*. The computational complexity of the depthwise convolution can be expressed as

(1) $$C_{depthwise}=H\times W\times K^{2}\times C_{in}$$

where *H* and *W* are the height and width of the feature map, *K* is the kernel size, and *C_in_* is the number of input channels.

The expansion convolution adjusts the number of input channels using pointwise convolution, expanding the channel count of the input feature map to provide a richer feature representation. In this study, the activation function in this stage is modified to the SiLU activation function, enhancing the nonlinear expressiveness of the extracted features. The formula for the SiLU activation function is as follows:

(2) $$f(x)=\frac{x}{1+e^{-x}}$$

The middle stage depthwise convolution is primarily used for further feature extraction from the expanded feature map. Whether the middle depthwise convolution is enabled depends on the settings of the intermediate convolution kernels. After the depthwise convolution, the output feature map undergoes channel shuffling, where the channel information of different convolution groups is exchanged, improving the model’s ability to extract and represent features. Channel shuffling mainly consists of group reshaping, permutation operations, and flattening operations.

Projection convolution primarily functions to reduce the dimensionality of the input features, which helps reduce model complexity and improve computational efficiency. The extracted features are then processed by the SE attention mechanism, allowing the model to focus more on the feature channels that contribute to wheat spike recognition. The computational steps of the SE module are as follows:

(3) $$x_{SE}=\sigma \left(\frac{1}{N}\sum_{i=1}^{N}x_{i}\right)\cdot x$$

where σ is the sigmoid activation function, *N* are learnable weights, and $x_{i}$ is the input feature map. If the stride is set to 1 and the input and output channels are the same, the module performs a residual connection by adding the input feature map x to the output feature map. This ensures stable gradient propagation within the network.

#### 2.3.2. Lightweight Downsampling Module

**Figure 5:**
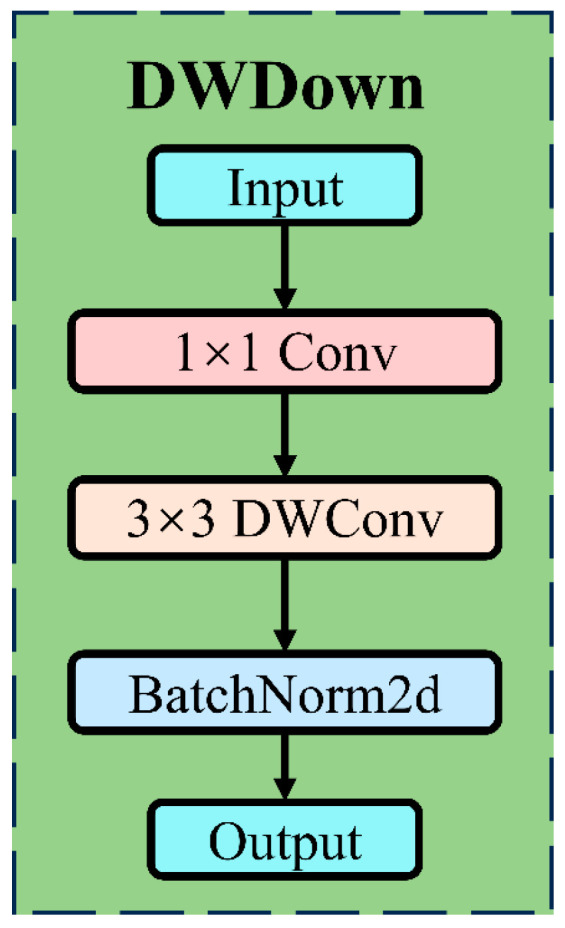
Structure of the DWDown module.

The downsampling module plays a crucial role in object detection networks. Downsampling is achieved through convolutional layers, typically using convolution operations with a stride of 2 to reduce high-resolution feature maps to lower resolutions, facilitating fusion with smaller-scale feature maps. At the same time, the reduction in feature map size helps decrease the model’s computational load and memory requirements. To improve computational efficiency and feature extraction capability, this study proposes a lightweight downsampling module named DWDown. The structure of the DWDown module is shown in Figure 5.

The DWDown module designed in this study consists of three main steps: channel adjustment, feature extraction, and feature normalization. Channel adjustment uses 1 × 1 convolutions to reduce the dimensionality of the input features. Feature extraction employs depthwise convolution to extract and enhance the features. Feature normalization applies batch normalization to standardize the output features, further improving the model’s convergence speed and stability. The normalization process can be expressed by the following formula:

(4) $$\hat{x}=\frac{x_{\mathrm{dw}}-\mu }{\sqrt{\sigma^{2}+\epsilon }}\cdot \gamma +\beta$$

where μ and $\sigma^{2}$ represent the mean and variance of the mini-batch, respectively, and γ and β are learnable parameters.

#### 2.3.3. Spatial Pyramid Pooling Fast Pyramid Module

**Figure 6:**
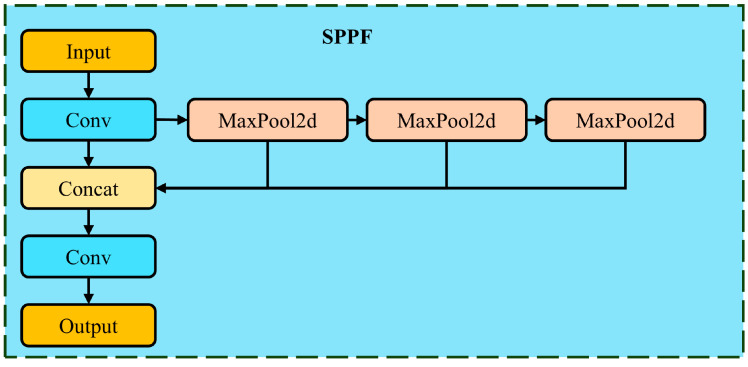
Structure of the SPPF module.

The Spatial Pyramid Pooling Fast (SPPF) module is used in object detection networks to enhance feature extraction, particularly for handling targets at different scales. The structure of the SPPF module is shown in Figure 6. It performs multi-scale pooling on the input feature map to extract a broader range of features while maintaining computational efficiency [44,45]. Specifically, the SPPF module first reduces the number of channels in the input feature map by half, then performs three separate max-pooling operations at different scales and concatenates the results with the original feature map. Finally, a convolutional layer is used to integrate these multi-scale features into a unified feature map.

#### 2.3.4. Lightweight Detect Head

**Figure 7:**
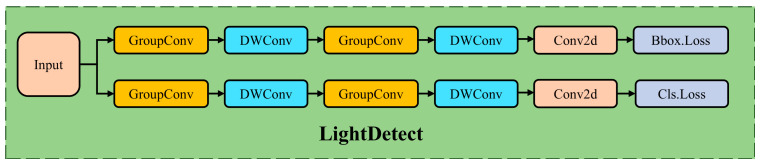
Module structure of lightweight detection head.

In this study, a new lightweight detection head (LightDetect) is designed based on the YOLOv8 detection head. The aim of this design is to reduce the computational burden of the model while maintaining detection performance. The improvement combines group convolutions and depthwise separable convolutions. The structure of the lightweight detection head is shown in Figure 7. LightDetect achieves efficient and accurate object detection through a carefully designed network structure, which consists of two main branches: one for bounding box regression prediction and another for class classification prediction.

In the regression branch, the detection head first uses group convolution to extract features. This operation reduces the number of parameters and computation by grouping while still maintaining effective feature extraction [46,47]. Next, the branch uses depthwise separable convolutions to further extract and merge features. This convolution method reduces computational complexity by separating depthwise convolutions from pointwise convolutions [48,49]. Finally, the branch outputs the predicted bounding box values using a 1 × 1 convolution layer. These predictions are based on anchor boxes, enabling the model to predict object locations.

The classification branch also starts with group convolution for feature extraction and enhancement. Then, depthwise separable convolutions refine the feature map further. Finally, the classification branch uses another 1 × 1 convolution layer to output class prediction values, reflecting the probability distribution of different classes.

LightDetect also includes a dynamic feature fusion module that effectively merges information across feature maps of different scales, enhancing the model’s ability to detect objects of varying sizes. During the prediction phase, the outputs from both branches are combined and decoded to obtain the final bounding boxes and class probabilities. In training mode, the detection head returns raw, unprocessed predictions, while in evaluation mode, additional post-processing steps are applied, such as non-maximum suppression and class probability threshold filtering, to output the final detection results.

#### 2.3.5. Loss Function

In the training process of object detection algorithms, the Intersection over Union (IoU) loss function is an important metric for evaluating the overlap between the predicted bounding box and the ground truth bounding box. A higher IoU value indicates a greater overlap between the predicted box and the ground truth, suggesting that the model performs well in terms of object localization. However, IoU only considers the ratio of intersection to union and does not reflect the spatial relationship between the predicted and actual bounding boxes. Furthermore, when there is no intersection between the predicted box and the actual object box, IoU is zero, which provides no effective gradient information for model training.

Therefore, based on IoU, the Complete Intersection over Union (CIOU) loss function, which combines center point distance and aspect ratio difference, is more suitable for training wheat spike detection models. CIOU more accurately measures the similarity between two bounding boxes with irregular shapes and sizes. The introduction of penalty terms for center point distance and aspect ratio ensures that the model more effectively constrains the position and shape of the predicted box under complex scenes and occluded objects. As a result, this study selects CIOU as the loss function for training. The calculation formula for the CIOU loss function is as follows:

(5) $$\mathrm{CIOU}=\mathrm{IoU}-\frac{r^{2}(b,b_{gt})}{c^{2}}-av$$

where $\rho^{2}(b,b_{gt})$ is the distance between the centers of the predicted and ground truth boxes, *c* is the diagonal length of the smallest enclosing box, *v* is the penalty term for aspect ratio, and *α* is the balancing parameter.

#### 2.3.6. Wheat Spike Detection Network Structure

**Figure 8:**
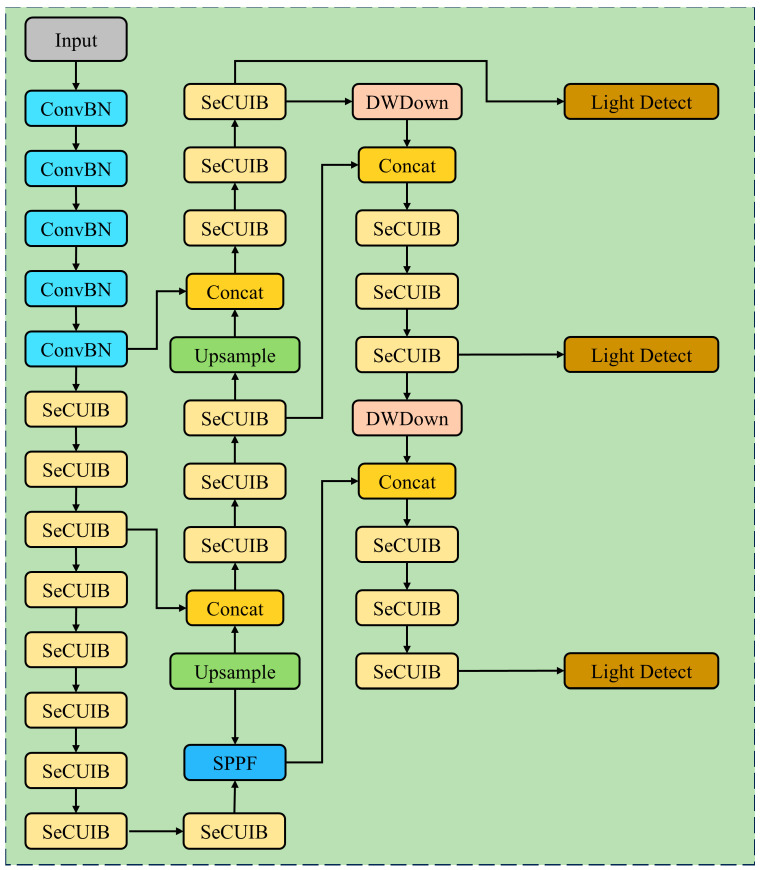
Structure diagram of wheat spike detection network.

This study aims to optimize the performance of spike extraction and detection tasks by constructing a new lightweight wheat spike detection network, LGWheatNet, based on multiple modules. The structure of the lightweight wheat spike detection network designed in this study is shown in Figure 8.

As seen in Figure 8, we selected a small-scale version of MobileNetV4 as the base network structure to ensure both the lightweight nature and efficiency of the model. We also optimized the UIB module and constructed a new SeCUIB module to rebuild the backbone network, combining it with SPPF to enhance the feature recognition and extraction capabilities for wheat spikes. The SeCUIB and DWDown modules were used to build the FPN + PAN neck network. Finally, a lightweight head network, LightDetect, was built by combining grouped convolutions and depthwise convolutions.

### 2.4. Experimental Setup

#### 2.4.1. Experimental Environment

The experimental environment for this study is configured as follows: a computer equipped with an NVIDIA A16 GPU, which has a floating point operation rate of 2.19 TFLOPS per second, 15 GB of GPU memory, and a bandwidth of 200.03 GB/s. The experimental computer supports 16 channels and provides a bandwidth of 31.50 GB/s through PCIE. In addition, the computer is equipped with 3 Intel(R) Xeon(R) Gold 5318Y CPUs running at 2.10 GHz and 14 GB of memory. The storage is of the LOGICAL VOLUME type, with a bandwidth of 431.04 MB/s and 100 GB of available space. The operating system is Ubuntu 20.04, and the environment includes Pytorch 2.1.1, Python 3.10, CUDA 11.8, cuDNN 8, and NVCC. For model training, this study employs SciPy version 1.14.1, Torchaudio version 2.1.1, Torchvision version 0.16.1, and Transformers version 4.47.0. In the domain of image processing and video analysis, OpenCV-Python version 4.9.0.80 and NumPy version 1.26.4 are utilized. The selection of these libraries ensures efficient and reliable performance in both model training and data processing.

#### 2.4.2. Experimental Parameter Settings

In this study, the model’s iteration count during training was set to epoch = 300, with the initial learning rate set to lr0 = 0.01. A learning rate decay factor (lrf = 0.01) was applied for gradual reduction to avoid overfitting and improve training performance. The momentum was set to 0.937 to accelerate convergence and increase stability during training. Weight decay was set to 0.0005 for regularization to prevent overfitting. To ensure a smooth model startup, a warm-up phase (warmup_epochs) of 3 epochs was employed, with the momentum during the warm-up set to 0.8 and the bias learning rate (warmup_bias_lr) set to 0.1.

### 2.5. Evaluation Metrics

To comprehensively evaluate the performance of different models in wheat spike detection tasks, this study employs several key metrics: Precision (P), Recall (R), Mean Average Precision (mAP50), and Extended Mean Average Precision (mAP50-95). P assesses the proportion of true positive samples among all predictions that were classified as positive. It directly reflects the model’s ability to predict positive classes accurately. R measures the proportion of true positive samples that were correctly identified as positive out of all actual positive samples, indicating the model’s ability to detect all positive classes. mAP50 calculates the average detection precision across all categories at an Intersection over Union (IoU) threshold of 0.5, assessing the overall performance of the model. mAP50-95 calculates the average precision (AP) for IoU thresholds ranging from 0.5 to 0.95 with a step size of 0.005, providing a comprehensive evaluation of the model’s performance across different levels of matching strictness. The specific calculation formulas are as follows:

(6) $$P=\frac{TP}{TP+FP}$$

(7) $$R=\frac{TP}{TP+FN}$$

(8) $$mAP50=\frac{1}{n}\sum_{i=1}^{n}AP_{i}$$

(9) $$mAP50-95=\frac{1}{n}\sum_{i=1}^{n}\frac{1}{91}\sum_{j=1}^{91}AP_{i,j}$$

where TP denotes true positives, FP denotes false positives, and FN denotes false negatives. The formula for mAP50 is the average precision at an IoU threshold of 0.5 for each class, and for mAP50-95, the AP values are averaged over IoU thresholds from 0.5 to 0.95, with 91 points computed for each threshold, spaced by 0.005.

To evaluate the performance of different models in wheat spike counting, this study uses RMSE, MAE, MSE, and R^2^ to assess the practical application performance of each model. The specific formulas are as follows:

(10) $$R^{2}=\frac{{\left[\sum_{i=1}^{n}\left(P_{i}-\bar{P_{i}}\right)\left(T_{i}-\bar{T_{i}}\right)\right]}^{2}}{\sum_{i=1}^{n}{\left(P_{i}-\bar{P_{i}}\right)}^{2}\sum_{i=1}^{n}{\left(T_{i}-\bar{T_{i}}\right)}^{2}}$$

(11) $$RMSE=\sqrt{\frac{\sum_{i=1}^{n}{\left(T_{i}-P_{i}\right)}^{2}}{n}}$$

(12) $$\mathrm{MSE}=\frac{1}{n}\sum_{i=1}^{n}{\left(T_{i}-P_{i}\right)}^{2}$$

(13) $$MAE=\frac{\sum_{i-1}^{n}\left|T_{i}-P_{i}\right|}{n}$$

where $T_{i}$ is the true number of wheat spikes, $P_{i}$ is the detected number of wheat spikes, $\bar{T_{i}}$ is the mean of the true values, and $\bar{P_{i}}$ is the mean of the predicted values.

## 3. Experimental Results

### 3.1. Ablation Experiments

In this study, the baseline wheat spike detection network was built using the small version of the MobileNetV4 network combined with SPPF as the base backbone, along with an FPN + PAN neck network reconstructed by the UIB module and the YOLOv8 detection head (Detect) module. To specifically analyze the roles of SeCUIB, DWDown, and LightDetect in the wheat spike detection task, ablation experiments were conducted to evaluate the performance of these modules within the network. The results of these ablation experiments are shown in Table 2.

As shown in Table 2, the baseline model constructed in this study achieves P = 0.941, R = 0.904, mAP50 = 0.960, and mAP50-95 = 0.712. Upon the introduction of the SeCUIB module, the model’s P, R, mAP50, and mAP50-95 improved by 0.8%, 1.2%, 0.4%, and 1.7%, respectively. The addition of the SeCUIB module slightly increased the model’s parameter count and computational load, but this increase is justified by the improvements in model performance. Subsequently, the inclusion of the DWDown module boosted R to 0.910 and mAP50-95 to 0.714 while reducing the model’s parameter count and computational load. The DWDown module not only decreased the computational burden and reduced parameters but also contributed to a certain degree of optimization in model performance. Finally, the introduction of LightDetect resulted in a slight improvement in model accuracy compared to the baseline model. The parameter count, GFLOPs, and weight size were significantly reduced, with reductions of 18.390%, 26.471%, and 15.556%, respectively. LightDetect alleviated the model’s computational load while maintaining its detection performance.

When the DWDown and LightDetect modules were used together, the model performance saw a notable improvement compared to using either DWConv or LightDetect modules individually. At the same time, the model’s parameter count, GFLOPs, and weight size all reached their lowest points, with reductions of 21.241%, 27.941%, and 17.778%, respectively, compared to the baseline model. The introduction of both DWDown and LightDetect demonstrated that they not only reduce the model’s computational burden but also contribute positively to enhancing model accuracy. When both SeCUIB and LightDetect modules were introduced, the model’s computational complexity fell between the individual computational complexities of SeCUIB and LightDetect. Similarly, when both SeCUIB and DWConv modules were used together, the model’s accuracy improved significantly, with computational costs also falling between the two modules. These results effectively demonstrate the advantage of SeCUIB in enhancing model accuracy. While it did not further reduce the model’s size, its contribution to performance improvement was significant. Finally, when all three modules were integrated, the model achieved P = 0.956, R = 0.921, mAP50 = 0.967, and mAP50-95 = 0.747, showing the best performance. Additionally, the model was further optimized in terms of lightweight characteristics, with a parameter count of 1,698,529, GFLOPs of 5.0, and a weight size of 3.9 MB. These results not only demonstrate the potential of the module combination to enhance model performance but also highlight their significant advantages in optimizing computational efficiency.

In conclusion, the integration of the SeCUIB, DWDown, and LightDetect modules not only improved detection accuracy but also optimized computational efficiency and parameter count. This makes the model highly suitable for deployment on resource-constrained devices. The collaborative effect of these modules makes the LGWheatNet model both highly efficient and practical for wheat spike detection tasks, offering great deployment potential in real-world applications.

### 3.2. Experimental Results of the Wheat Spike Detection Model

**Figure 9:**
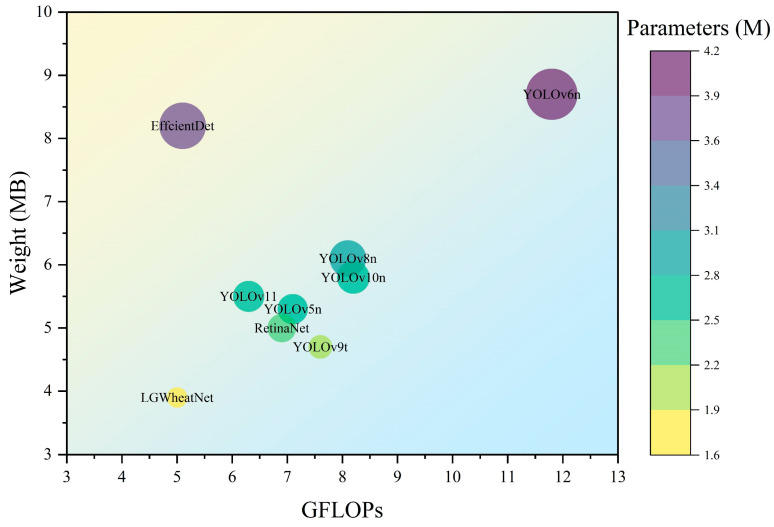
Comparison of number of parameters, weights, and GFLOPs for different models.

**Figure 10:**
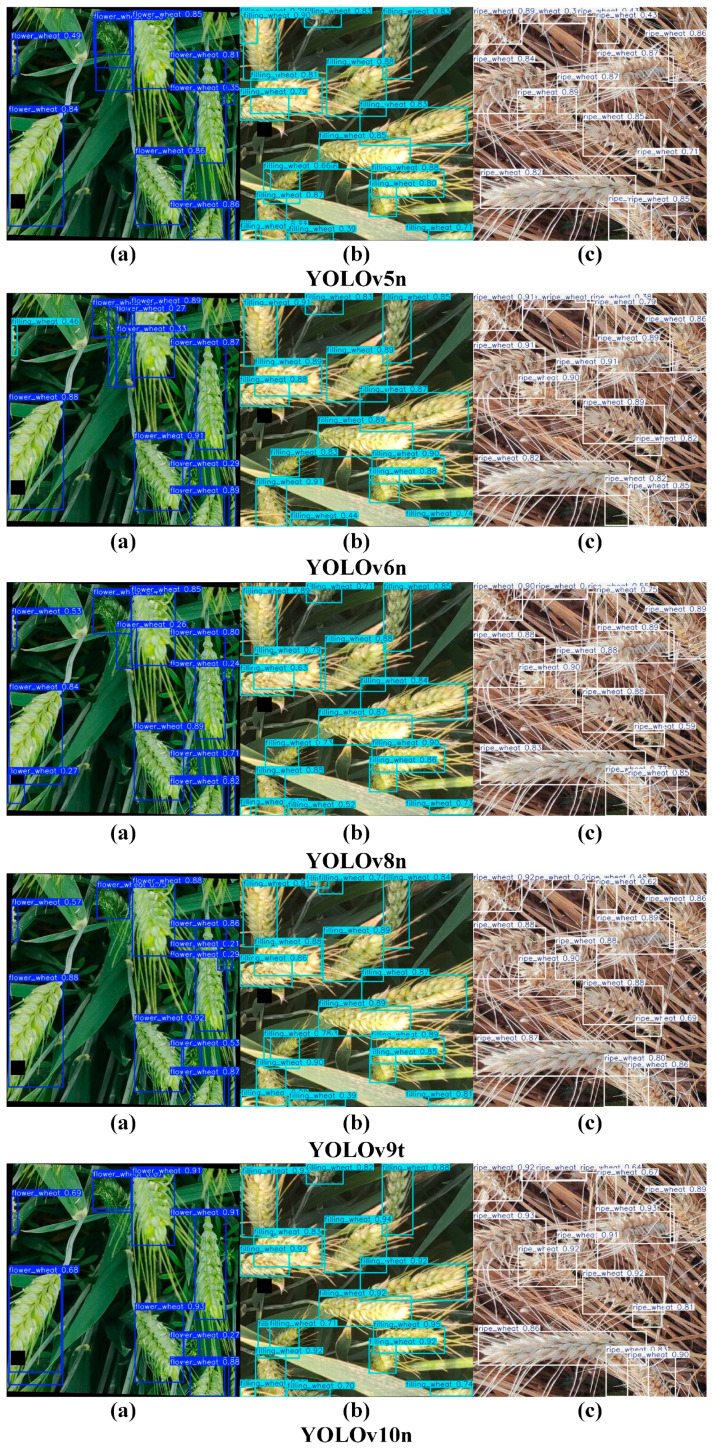

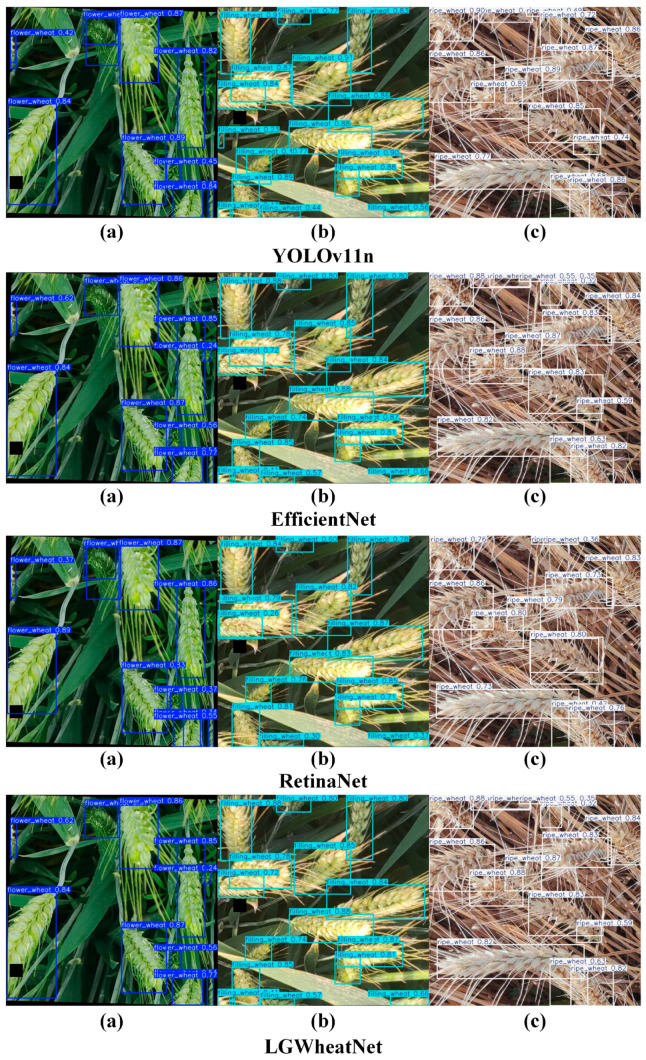
Results of wheat spike detection by different models: (**a**–**c**) wheat spike images from the flowering, grain-filling, and maturity stages, respectively.

This study constructed a multi-growth-stage wheat spike dataset based on three key growth stages of wheat: flowering, filling, and maturity. The aim was to enhance detection accuracy across these different growth stages. A novel network module, SeCUIB, was developed and integrated with lightweight downsampling modules (DWDown, SPPF) and a lightweight detection head (LightDetect) to design a network specifically for wheat spike detection named LGWheatNet. To comprehensively evaluate the performance of LGWheatNet, several comparison models from the YOLO series (YOLOv5, YOLOv6, YOLOv8, YOLOv9t, YOLOv10, YOLOv11), EfficientDet, and RetinaNet were selected. Table 3 presents the experimental results for various models while Figure 9 compares the parameter sizes and Figure 10 illustrates the detection results.

The experimental results demonstrated that LGWheatNet outperformed several mainstream detection models in key metrics such as Precision (P), Recall (R), mAP50, and mAP50-95. Overall, LGWheatNet achieved a P of 0.956, R of 0.921, mAP50 of 0.967, and mAP50-95 of 0.747, significantly surpassing YOLOv5n, YOLOv6n, YOLOv8n, and YOLOv10n. In wheat spike detection across different growth stages, LGWheatNet exhibited remarkable performance in each stage. During the flowering stage, LGWheatNet achieved a P of 0.937, R of 0.880, mAP50 of 0.943, and mAP50-95 of 0.662, outperforming YOLOv5n, YOLOv6n, YOLOv8n, and YOLOv10n, whose mAP50-95 values were 0.661, 0.662, 0.663, and 0.664, respectively. During the filling stage, LGWheatNet achieved a P of 0.957, R of 0.937, mAP50 of 0.975, and mAP50-95 of 0.785, with P and mAP50-95 surpassing all comparison models. In the maturity stage, LGWheatNet achieved a P of 0.973, R of 0.946, mAP50 of 0.982, and mAP50-95 of 0.794, maintaining its advantage over other models.

In addition to its accuracy advantages, LGWheatNet also excelled in computational efficiency. Specifically, LGWheatNet had 1,698,529 parameters and required 5.0 GFLOPs, nearly halving the parameters and computational load compared to YOLOv6n, which had 4,234,041 parameters and required 11.8 GFLOPs. Furthermore, YOLOv9t and YOLOv8n required 7.6 and 8.1 GFLOPs, respectively, exceeding the computational requirements of LGWheatNet, further highlighting LGWheatNet’s efficiency. Although EfficientDet required only 5.1 GFLOPs, its detection accuracy was significantly lower than LGWheatNet. RetinaNet, with 2,358,261 parameters and 6.9 GFLOPs, was lightweight in terms of computational cost but failed to match LGWheatNet in detection accuracy. These comparisons illustrate that LGWheatNet not only achieves superior detection accuracy but also maintains high computational efficiency, making it particularly suitable for deployment in resource-constrained environments.

**Figure 11:**
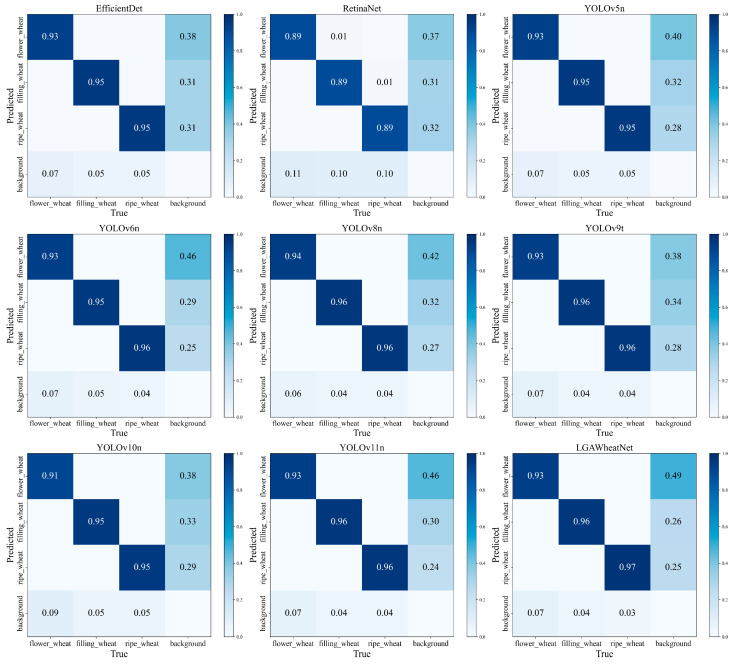
The regularized confusion matrix plot of the detection results of different models.

To provide a more comprehensive evaluation of the performance of different models, a confusion matrix was used in this study for further analysis. The confusion matrices for the different models are shown in Figure 11.

As seen in Figure 11, the LGWheatNet model performs excellently across different wheat growth stages. During the flowering stage, the recognition accuracy of wheat spikes was 0.930, slightly lower than YOLOv10n’s 0.94, but still highly competitive. During the grain filling stage, the recognition accuracy reached 0.960, on par with other models, with no significant difference. In the maturity stage, the recognition accuracy reached 0.970, the highest among all models, demonstrating LGWheatNet’s outstanding feature extraction capabilities. Additionally, LGWheatNet showed stable performance in minimizing background misidentifications. While misidentifications were slightly higher during the flowering stage, the misidentification rate in the grain filling and maturity stages was the lowest among all models, further verifying its precision in recognizing key targets and its effective suppression of interference information. Overall, LGWheatNet stands out in both accuracy and robustness, making it an excellent detection model.

### 3.3. The Validation of Model Validity

To validate the effectiveness of the LGWheatNet model, this study conducted verification analysis on the publicly available Global Wheat Head Detection (GWHD) dataset. The GWHD dataset, initially created in 2020, consists of 4700 RGB images sourced from platforms across seven countries/institutes, annotating 193,634 wheat spike targets [50]. Based on experiences from the 2020 competition on Kaggle, researchers identified areas for improvement in terms of dataset scale, spike diversity, and label reliability. To address these issues, the 2020 version was re-examined and re-annotated, with an expanded dataset that included 1722 additional images from five other countries/regions and 81,553 new annotations. In 2021, the research team released the updated GWHD2021 dataset [51]. This study trained LGWheatNet on the GWHD2021 dataset and compared its performance to related studies found in CNKI and Google Scholar to provide a more comprehensive evaluation. The experimental results of the studies collected by this research are shown in Table 4.

As shown in Table 4, LGWheatNet outperforms the comparison models in terms of overall performance in wheat spike detection tasks, exhibiting exceptional detection capabilities. LGWheatNet achieved an accuracy of 0.921, improving by 0.327% and 3.136% compared to the improved YOLOv5 (0.918) and RIA-SpikeNet (0.893), respectively, highlighting its superiority in classification accuracy. In terms of recall, LGWheatNet achieved a recall rate of 0.880, far higher than RIA-SpikeNet’s 0.714, indicating a more comprehensive detection ability for wheat spike targets and a significant reduction in false negatives. For the mAP50 metric, LGWheatNet reached 0.937, outperforming TPH-YOLO (0.916), the improved YOLOv5 (0.918), RT-WEDT (0.902), and RIA-SpikeNet (0.815) by 2.293%, 2.070%, 3.880%, and 14.969%, respectively. This demonstrates a significant advantage in detection accuracy across different target scales and complex scenarios.

Overall, the experimental results on GWHD2021 fully validate the effectiveness and robustness of LGWheatNet in wheat spike detection tasks, showcasing its strong generalization ability and potential for real-world applications.

### 3.4. Experimental Results of Wheat Spike Counting

**Figure 12:**
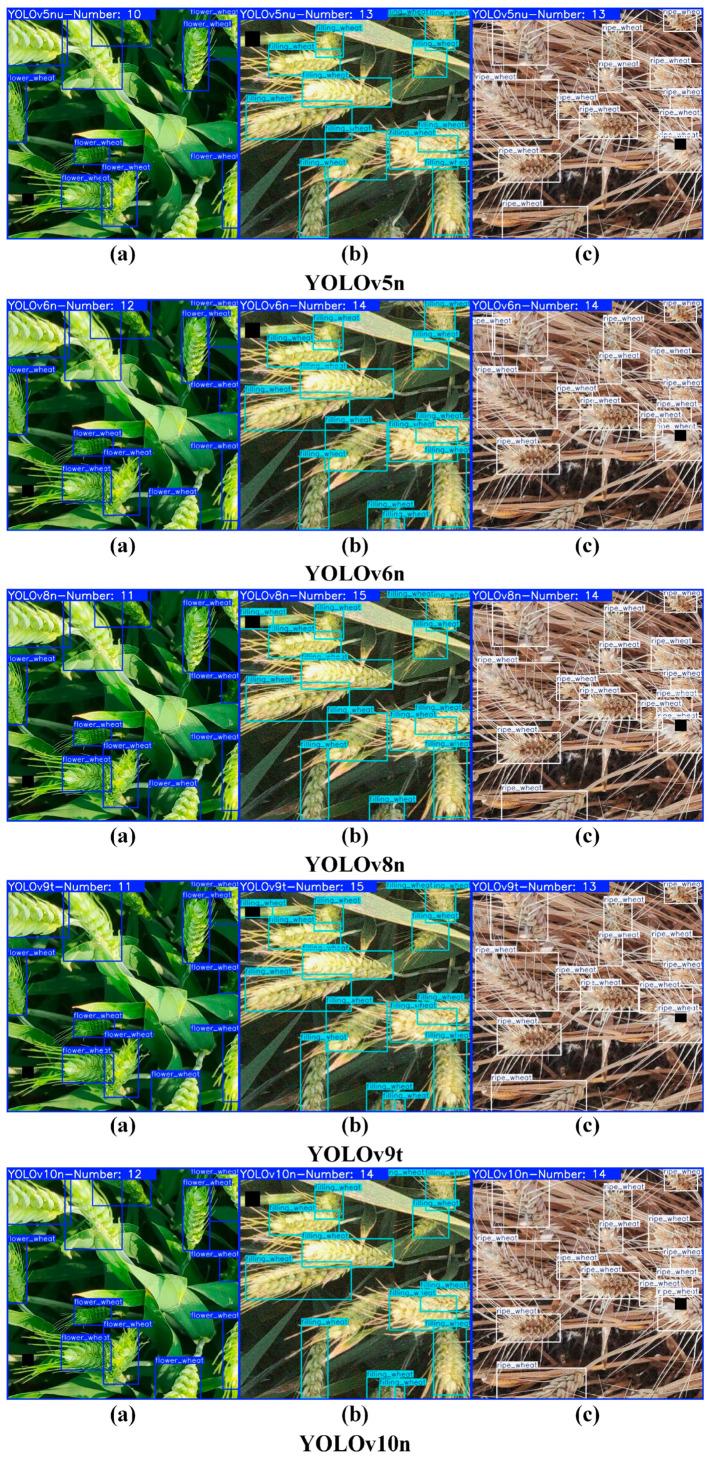

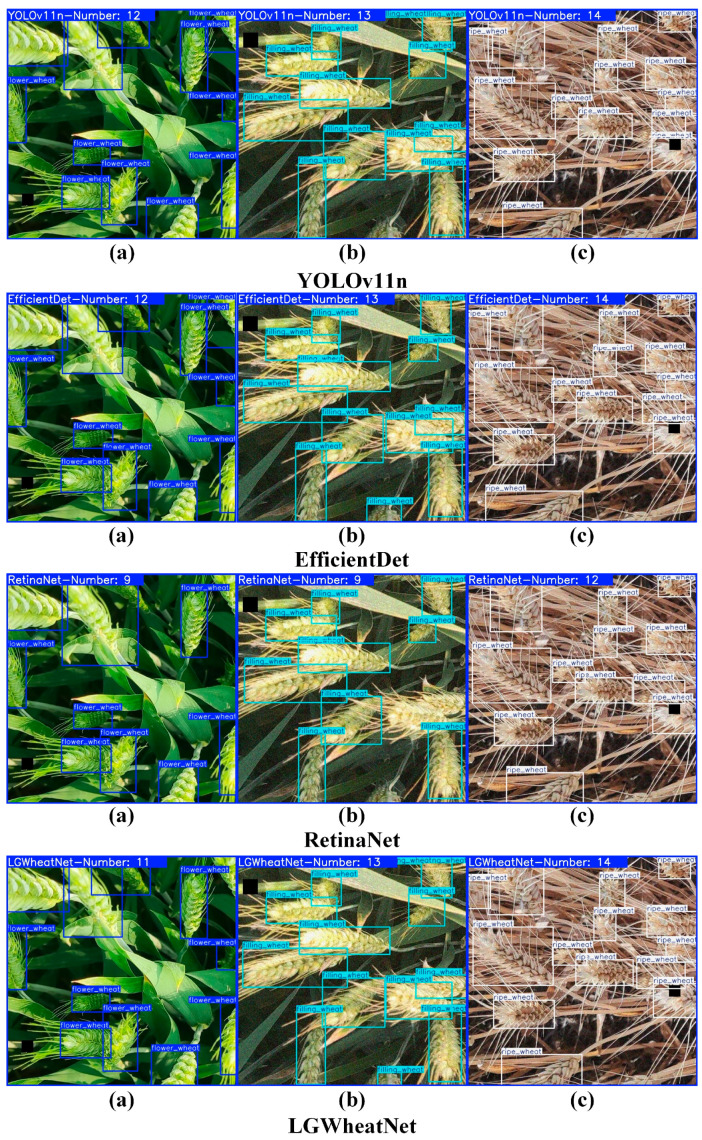
Wheat spike region counting results for different models: (**a**–**c**) wheat spike images from the flowering, grain-filling, and maturity stages, respectively.

In this investigation, the wheat spike detection model LGWheatNet, which we engineered, along with the YOLO series algorithms, EfficientDet, and Retinanet, showcased their precision in the experimental outcomes concerning the wheat spike area counting, as delineated in Table 5. The counting example diagrams for disparate models are presented in Figure 12.

The results demonstrate that LGWheatNet exhibits high prediction accuracy and stability in wheat spike region counting, achieving an RMSE of 2.205, an R^2^ of 0.913, an MAE of 1.407, and an MSE of 6.489. While LGWheatNet’s overall precision is slightly lower than YOLOv9t and YOLOv10n, it delivers robust performance across different growth stages. For the flowering stage, LGWheatNet achieved an RMSE of 4.007, an R^2^ of 0.963, an MAE of 2.604, and an MSE of 16.058, indicating high prediction accuracy. For the filling and maturity stages, LGWheatNet demonstrated lower error values and higher R^2^ scores, with RMSEs of 1.364 and 1.245, R^2^ values of 0.869 and 0.908, MAEs of 0.841 and 0.776, and MSEs of 1.859 and 1.550, respectively.

Although LGWheatNet shows some performance gaps in certain metrics when compared to some models, its performance is still sufficient to serve as a highly effective model for practical applications. Additionally, LGWheatNet has distinct advantages in terms of parameter count, floating-point operations, and model weight. Therefore, the LGWheatNet model developed in this study provides an efficient and reliable solution for wheat spike counting tasks in agricultural applications.

## 4. Extension and Application of LGWheatNet

**Figure 13:**
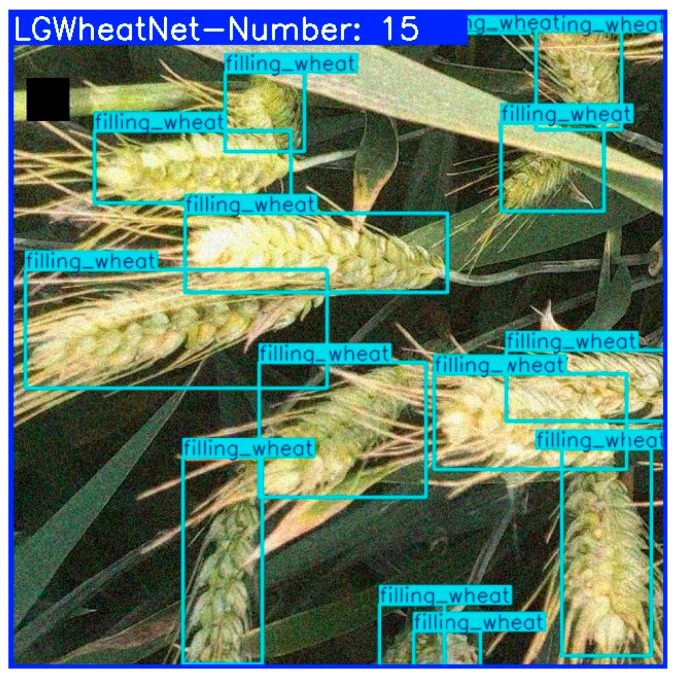
Experimental results of small-scale wheat ear inference for the LGWheatNet model.

**Figure 14:**
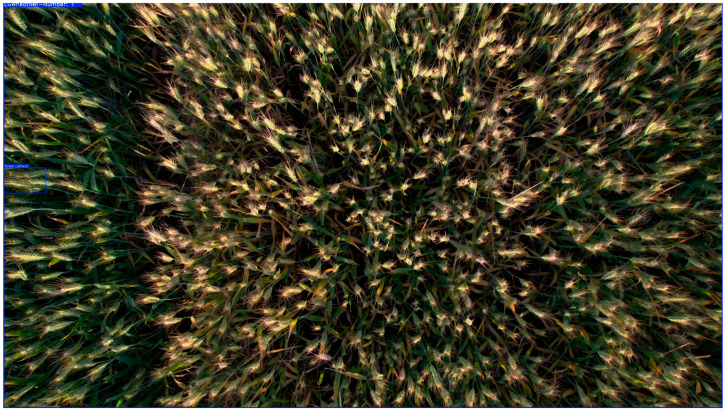
Experimental results of large-scale wheat ear inference for the LGWheatNet model.

LGWheatNet performs exceptionally well in small-scale wheat ear detection applications, with experimental results for small-scale wheat ear detection shown in Figure 13. In practical applications, drones are commonly used for large-scale wheat ear data collection. The results of direct inference for large-scale wheat ear counting using the wheat ear detection model are shown in Figure 14.

High-resolution images captured by drones, when directly processed by the model for inference, typically undergo compression first. However, image compression can result in the loss of small target details, such as those of wheat ears, making it difficult for the model to accurately detect these small-scale targets. To address this issue, this study proposes a strategy combining the wheat ear detection model with Slicing Aided Hyper Inference (SAHI) technology. By performing image slicing, this approach mitigates the feature loss caused by compression, thereby improving the detection accuracy for small targets.

In this study, the effectiveness of the SAHI framework in processing high-resolution, large-scale drone images was evaluated, with a focus on its performance in multi-scale wheat ear detection. The study integrated the self-constructed wheat ear detection model LGWheatNet with the SAHI framework, employing a slice-based inference strategy to accurately detect wheat targets in large-scale images.

**Figure 15:**
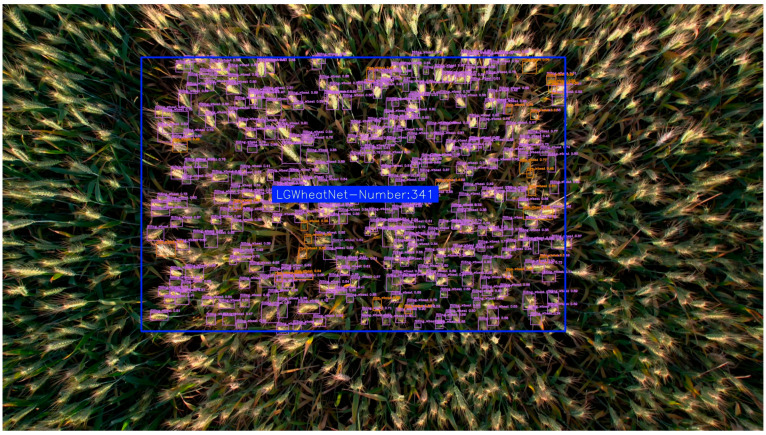
Experimental results of combining LGWheatNet with SAHI in single-region inference.

**Figure 16:**
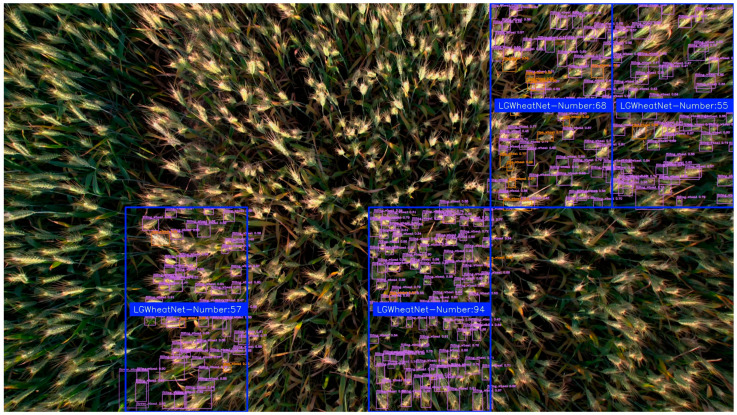
Experimental results of combining LGWheatNet with SAHI in multi-region inference.

In terms of experimental data collection and parameter settings, this study utilized the DJI Air 2S drone to collect data on wheat spikes in a field at a height of 2 m. Taking wheat spikes during the grain filling stage as an example, large-scale images of the wheat field were captured using this drone, ensuring high-resolution and comprehensive coverage of the image data. For the SAHI, the slice size was set to 256 × 256 pixels, with an overlap ratio of 0.1 along both the height and width, ensuring sufficient overlap between slices to improve the detection of edge areas. Experimental results for both single-region and multi-region wheat ear detection are shown in Figure 15 and Figure 16.

As shown in Figure 15 and Figure 16, this study integrated LGWheatNet with SAHI inference and achieved commendable performance in large-scale wheat spike detection with drone-collected images. Thus, combining LGWheatNet trained on small-scale data with SAHI inference enables effective detection of large-scale wheat spikes, yielding good results. This approach offers a valuable reference for model training and application in low-cost agricultural detection.

## 5. Inference Time of LGWheatNet Deployment on CPU and GPU

In this study, the LGWheatNet model, trained using the PyTorch framework, was exported to various formats, including PyTorch, TensorRT, TorchScript, OpenVINO, and ONNX, for deployment on both CPU and GPU platforms. The optimal deployment format was determined based on the fastest inference speed observed during model evaluation. The inference times for processing a single image under different deployment formats on CPU and GPU are presented in Table 6 and Table 7, respectively.

As shown in Table 6, for CPU-based inference, the native PyTorch model exhibited the longest inference time per image at 102.6 ms. Deploying the model in TorchScript format reduced the inference time by 17.641%. When exported to ONNX format, the inference speed further improved, achieving an inference time of 22.4 ms per image. The OpenVINO format demonstrated the fastest inference time on CPU, with an average inference time of 15.7 ms per image. This indicates that OpenVINO is the most suitable deployment format for CPU-based inference.

For GPU inference, inference times were significantly reduced compared to CPU, with an even greater disparity among formats. The PyTorch model achieved an inference time of 15.7 ms per image on GPU. After conversion to TensorRT, the inference time was reduced to 5.3 ms, representing a 66.242% reduction compared to the PyTorch format. In contrast, the OpenVINO and ONNX formats exhibited slower inference speeds, with inference times of 22.2 ms and 28.1 ms per image, respectively.

In summary, OpenVINO is recommended for CPU inference due to its superior speed. For GPU inference, TensorRT is the optimal choice, offering the fastest inference time. Additionally, TorchScript demonstrated strong performance on GPU, with an inference time second only to TensorRT. Therefore, for practical deployment, this study prioritizes the use of TensorRT and TorchScript formats to maximize the computational capabilities of NVIDIA GPUs. For models requiring CPU execution, OpenVINO is the preferred format to ensure efficient and stable inference.

## 6. Discussion

The LGWheatNet model proposed in this study demonstrated outstanding performance in the wheat spike detection task, surpassing several mainstream detection models in key metrics such as Precision, Recall, mAP50, and mAP50-95. Additionally, it displayed significant advantages in terms of parameter count and GFLOPs, highlighting its lightweight characteristics. These results suggest that the LGWheatNet model has vast potential for application in resource-constrained environments, such as mobile devices or edge computing platforms.

The SeCUIB module enhances feature representation through channel attention and shuffle operations. Zhao et al. [56] leveraged SE attention to mitigate noise interference in high-mobility vehicular networks with time-varying channels while emphasizing useful feature information. Das et al. [57] employed channel shuffle to facilitate cross-group feature information flow across channels, thereby enhancing feature recognition performance. The DWDown module effectively reduces spatial dimensions using depthwise convolution and channel adjustment. Meanwhile, the LightDetect detection head integrates grouped convolution and depthwise separable convolution, reducing computational overhead while maintaining performance. Zhang et al. [58] demonstrated that depthwise convolution significantly reduces model parameters and computational burden, leading to improved performance in their adjusted model. Similar conclusions were drawn in studies employing comparable methodologies. Collectively, these design elements contribute to the high performance and lightweight characteristics of the proposed model in wheat spike detection.

Experimental results indicate that LGWheatNet exhibits superior performance in spike inflorescence detection across different wheat growth stages, demonstrating strong generalization and adaptability. Notably, in the mature stage, LGWheatNet achieves exceptionally high accuracy and recall. The high accuracy in wheat spike counting during the mature stage can likely be attributed to the withering and shedding of leaves, which reduce background interference and enhance the model’s ability to distinguish wheat spikes. In contrast, the lower recognition accuracy during the grain-filling stage may result from the presence of willow catkins covering wheat spikes and the high local similarity of jointing-stage wheat spikes, which can lead to misclassification. The reduced performance in the flowering stage is likely due to the small size of some emerging spikes and partial occlusion by leaves, which hinders detection accuracy. In the wheat spike counting experiment, LGWheatNet demonstrated outstanding performance, rivaling larger models in accuracy. Additionally, this study integrates LGWheatNet with SAHI for detecting large-scale wheat spikes in drone-captured imagery. The combination of LGWheatNet and SAHI shows promising potential for applications in UAV-based wheat monitoring.

However, due to budget constraints, the study only collected data from one wheat variety in the research area, and the sample size remains limited. The model’s performance in final applications still lags behind some larger models. Furthermore, only a portion of the drone-collected data was used for the demonstration application, which serves as a preliminary step for further deployment. Therefore, in future research, we plan to continue expanding our dataset, optimizing and fine-tuning the model, and accelerating its practical application. Additionally, we will focus on building dedicated datasets for drone-collected data to further validate and enhance the performance of the LGWheatNet model. To further enhance the practical applicability of the model, we plan to deploy it on mobile devices in the future, enabling farmers to more conveniently identify and count wheat spikes.

## 7. Conclusions

This study developed a lightweight model, LGWheatNet, for detecting wheat spikes across different growth stages, including flowering, filling, and maturity. The model was able to detect wheat spikes efficiently at these key growth stages, with experimental results showing mAP50 values of 0.943, 0.975, and 0.982, respectively, for each stage, highlighting its outstanding detection accuracy, especially during the maturity stage. In the static wheat spike counting task, LGWheatNet demonstrated high accuracy with an RMSE of 2.205, an R^2^ of 0.913, an MAE of 1.407, and an MSE of 6.489. These metrics indicate that LGWheatNet provides stable counting results across different growth stages, making it suitable for static wheat yield estimation. Moreover, the integration of the SAHI strategy enables LGWheatNet to perform well in large-scale wheat spike detection. This can serve as a useful reference for the broader application of models trained on small-scale data. The high performance of the LGWheatNet model is attributed to the effective integration of modules such as SeCUIB, LightDetect, SPPF, and DWDown, which not only maintain the model’s lightweight nature but also enable high-precision spike detection. As a result, LGWheatNet offers high practical value and can provide efficient and accurate technical support for wheat yield assessment and field monitoring. In future research, we plan to collect data from more wheat varieties to further optimize the LGWheatNet model’s structure in order to improve recognition accuracy. Additionally, its application potential in a wider range of agricultural scenarios should be explored to advance agricultural technology and provide innovative solutions for the agricultural sector.

## Figures and Tables

**Table 1:** Weather Conditions on Data Collection Dates.

Date	Temperature	Weather	Wind Direction and Speed
21 April 2024	15–26°	Cloudy to Sunny	NE Wind, Level 1
13 May 2024	19–30°	Sunny	S Wind, Level 3
27 May 2024	16–31°	Cloudy to Sunny	NE Wind, Level 4

**Table 2:** The results of ablation experiments for target detection of wheat spikes.

SeCUIB	DWDown	LightDetect	P	R	mAP50	mAP50-95	Parameters	Gflops	Weight (MB)
-	-	-	0.941	0.904	0.960	0.712	2,058,649	6.8	4.5
√	-	-	0.949	0.916	0.964	0.729	2,135,809	6.9	4.7
-	√	-	0.940	0.910	0.960	0.714	1,999,945	6.7	4.4
-	-	√	0.942	0.906	0.960	0.715	1,680,073	5.0	3.8
-	√	√	0.949	0.912	0.962	0.727	1,621,369	4.9	3.7
√	√	-	0.950	0.920	0.966	0.741	2,077,105	6.8	4.6
√	-	√	0.948	0.914	0.965	0.739	1,757,233	5.0	4.0
√	√	√	0.956	0.921	0.967	0.747	1,698,529	5.0	3.9

**Table 3:** Detection Accuracy of Wheat Spikes Using Various Models.

Models	Class	P	R	mAP50	mAP50-95
YOLOv5n	all	0.941	0.909	0.965	0.713
	flower wheat	0.932	0.881	0.948	0.661
	filling wheat	0.939	0.925	0.971	0.741
	ripe wheat	0.954	0.922	0.975	0.737
YOLOv6n	all	0.950	0.918	0.965	0.733
	flower wheat	0.936	0.888	0.945	0.662
	filling wheat	0.950	0.933	0.972	0.766
	ripe wheat	0.964	0.934	0.978	0.771
YOLOv8n	all	0.949	0.914	0.967	0.728
	flower wheat	0.942	0.883	0.950	0.663
	filling wheat	0.943	0.930	0.971	0.758
	ripe wheat	0.961	0.930	0.979	0.762
YOLOv9t	all	0.956	0.922	0.968	0.732
	flower wheat	0.951	0.888	0.954	0.672
	filling wheat	0.949	0.941	0.974	0.765
	ripe wheat	0.967	0.937	0.977	0.760
YOLOv10n	all	0.941	0.915	0.963	0.729
	flower wheat	0.932	0.875	0.944	0.664
	filling wheat	0.937	0.934	0.969	0.760
	ripe wheat	0.954	0.937	0.975	0.762
YOLOv11	all	0.953	0.918	0.969	0.732
	flower wheat	0.940	0.891	0.950	0.666
	filling wheat	0.949	0.934	0.976	0.766
	ripe wheat	0.970	0.929	0.982	0.765
EfficientDet	all	0.941	0.894	0.950	0.688
	flower wheat	0.938	0.867	0.931	0.633
	filling wheat	0.935	0.905	0.956	0.714
	ripe wheat	0.950	0.909	0.962	0.717
Retinanet-MobileNetv3	all	0.896	0.832	0.915	0.621
	flower wheat	0.889	0.825	0.912	0.594
	filling wheat	0.888	0.836	0.912	0.637
	ripe wheat	0.912	0.834	0.921	0.631
LGWheatNet	all	0.956	0.921	0.967	0.747
	flower wheat	0.937	0.88	0.943	0.662
	filling wheat	0.957	0.937	0.975	0.785
	ripe wheat	0.973	0.946	0.982	0.794

**Table 4:** The experimental results of model validity validation.

Model	P	R	mAP50	mAP50-95
TPH-YOLO [52]			0.916	
Improved YOLOv5 [53]	0.918		0.918	
RT-WEDT [54]			0.902	
RIA-SpikeNet [55]	0.893	0.714	0.815	
LGWheatNet	0.921	0.880	0.937	0.542

**Table 5:** Accuracy of wheat spike region counting for different models.

Model	Data	RMSE	MAE	MSE	R^2^
EfficientDet	all	2.803	1.961	9.447	0.827
	flowerwheat	4.587	3.325	21.039	0.952
	fillingwheat	1.877	1.246	3.522	0.752
	ripewheat	1.945	1.313	3.781	0.776
LGWheatNet	all	2.205	1.407	6.489	0.913
	flowerwheat	4.007	2.604	16.058	0.963
	fillingwheat	1.364	0.841	1.859	0.869
	ripewheat	1.245	0.776	1.550	0.908
RetinaNet	all	2.758	1.748	10.425	0.873
	flowerwheat	5.131	3.019	26.331	0.940
	fillingwheat	1.532	1.079	2.348	0.835
	ripewheat	1.611	1.145	2.595	0.846
YOLOv10n	all	1.760	1.160	3.556	0.923
	flowerwheat	2.715	1.812	7.370	0.983
	fillingwheat	1.335	0.877	1.783	0.874
	ripewheat	1.230	0.791	1.514	0.910
YOLOv11n	all	2.768	1.748	10.887	0.881
	flowerwheat	5.307	3.357	28.162	0.935
	fillingwheat	1.560	1.003	2.435	0.829
	ripewheat	1.437	0.883	2.064	0.878
YOLOv5n	all	2.554	1.696	8.512	0.879
	flowerwheat	4.549	3.071	20.695	0.952
	fillingwheat	1.582	1.003	2.501	0.824
	ripewheat	1.530	1.013	2.341	0.861
YOLOv6n	all	2.471	1.560	8.899	0.908
	flowerwheat	4.830	3.013	23.325	0.946
	fillingwheat	1.419	0.923	2.013	0.858
	ripewheat	1.166	0.743	1.359	0.919
YOLOv8n	all	2.598	1.735	9.014	0.881
	flowerwheat	4.726	3.136	22.331	0.949
	fillingwheat	1.569	1.038	2.460	0.827
	ripewheat	1.501	1.031	2.252	0.867
YOLOv9t	all	2.108	1.424	5.367	0.896
	flowerwheat	3.438	2.364	11.818	0.973
	fillingwheat	1.689	1.090	2.854	0.799
	ripewheat	1.196	0.819	1.430	0.915

**Table 6:** Inference speed of LGWheatNet on CPU across different formats.

Deployment Format	Inference Time per Image (ms)
pytorch	102.6
torchscript	84.5
openvino	15.7
onnx	22.4

**Table 7:** Inference speed of LGWheatNet on GPU across different formats.

Deployment Format	Inference Time per Image (ms)
pytorch	15.7
TensorRT	5.3
openvino	22.2
torchscript	9.4
onnx	28.1

## Data Availability

Because of the lab’s data confidentiality requirements, the raw data supporting the conclusions of this article will be made available by the authors on request.

## References

[1] Pequeno D.N., Hernandez-Ochoa I.M., Reynolds M., Sonder K., MoleroMilan A., Robertson R.D., Lopes M.S., Xiong W., Kropff M., Asseng S. (2021). Climate impact and adaptation to heat and drought stress of regional and global wheat production. Environ. Res. Lett..

[2] Li Z., Zhu Y., Sui S., Zhao Y., Liu P., Li X. (2024). Real-time detection and counting of wheat ears based on improved YOLOv7. Comput. Electron. Agric..

[3] Ma J., Li Y., Liu H., Wu Y., Zhang L. (2022). Towards improved accuracy of UAV-based wheat ears counting: A transfer learning method of the ground-based fully convolutional network. Expert Syst. Appl..

[4] Gupta A., Anand R., Sindhwani N., Mittal M., Dahiya A. (2024). Performance and Accuracy Enhancement of Machine Learning & IoT-based Agriculture Precision AI System. SN Comput. Sci..

[5] Xu X., Li H., Yin F., Xi L., Qiao H., Ma Z., Shen S., Jiang B., Ma X. (2020). Wheat ear counting using K-means clustering segmentation and convolutional neural network. Plant Methods.

[6] Geng Q., Zhang H., Gao M., Qiao H., Xu X., Ma X. (2024). A rapid, low-cost wheat spike grain segmentation and counting system based on deep learning and image processing. Eur. J. Agron..

[7] Wenchao L., Bin L., Dayu P., Yong Z., Chunhua Y., Cheng W. (2016). Synchronous measurement of wheat ear length and spikelets number based on image processing. J. Chin. Agric. Mech.

[8] Padhiary M., Saha D., Kumar R., Sethi L.N., Kumar A. (2024). Enhancing Precision Agriculture: A Comprehensive Review of Machine Learning and AI Vision Applications in All-Terrain Vehicle for Farm Automation. Smart Agric. Technol..

[9] Sadeghi-Tehran P., Virlet N., Ampe E.M., Reyns P., Hawkesford M.J. (2019). DeepCount: In-field automatic quantification of wheat spikes using simple linear iterative clustering and deep convolutional neural networks. Front. Plant Sci..

[10] Zhou C., Liang D., Yang X., Yang H., Yue J., Yang G. (2018). Wheat ears counting in field conditions based on multi-feature optimization and TWSVM. Front. Plant Sci..

[11] Fernandez-Gallego J.A., Buchaillot M.L., Aparicio Gutiérrez N., Nieto-Taladriz M.T., Araus J.L., Kefauver S.C. (2019). Automatic wheat ear counting using thermal imagery. Remote Sens..

[12] Hong Q., Liu W., Zhu Y., Ren T., Shi C., Lu Z., Yang Y., Deng R., Qian J., Tan C. (2024). CTHNet: A network for wheat ear counting with local-global features fusion based on hybrid architecture. Front. Plant Sci..

[13] Liu L., Ouyang W., Wang X., Fieguth P., Chen J., Liu X., Pietikäinen M. (2020). Deep learning for generic object detection: A survey. Int. J. Comput. Vis..

[14] Quan L., Feng H., Lv Y., Wang Q., Zhang C., Liu J., Yuan Z. (2019). Maize seedling detection under different growth stages and complex field environments based on an improved Faster R–CNN. Biosyst. Eng..

[15] Xia Y., Che T., Meng J., Hu J., Qiao G., Liu W., Kang J., Tang W. (2024). Detection of surface defects for maize seeds based on YOLOv5. J. Stored Prod. Res..

[16] Ukwuoma C.C., Zhiguang Q., Bin Heyat M.B., Ali L., Almaspoor Z., Monday H.N. (2022). Recent advancements in fruit detection and classification using deep learning techniques. Math. Probl. Eng..

[17] Razavi M., Mavaddati S., Koohi H. (2024). ResNet deep models and transfer learning technique for classification and quality detection of rice cultivars. Expert Syst. Appl..

[18] Ren S., He K., Girshick R., Sun J. (2016). Faster R-CNN: Towards real-time object detection with region proposal networks. IEEE Trans. Pattern Anal. Mach. Intell..

[19] Math R.M., Dharwadkar N.V. (2023). Deep learning and computer vision for leaf miner infestation severity detection on muskmelon (Cucumis melo) leaves. Comput. Electr. Eng..

[20] Shao Y., Guan X., Xuan G., Liu H., Li X., Gu F., Hu Z. (2024). Detection of Straw Coverage under Conservation Tillage Based on an Improved Mask Regional Convolutional Neural Network (Mask R-CNN). Agronomy.

[21] Li L., Hassan M.A., Yang S., Jing F., Yang M., Rasheed A., Wang J., Xia X., He Z., Xiao Y. (2022). Development of image-based wheat spike counter through a Faster R-CNN algorithm and application for genetic studies. Crop J..

[22] Wang C.-Y., Liao H.-Y.M. (2024). YOLOv1 to YOLOv10: The fastest and most accurate real-time object detection systems. arXiv.

[23] Liu W., Anguelov D., Erhan D., Szegedy C., Reed S., Fu C.-Y., Berg A.C. Ssd: Single shot multibox detector. Proceedings of the Computer Vision–ECCV 2016: 14th European Conference.

[24] Tan M., Le Q. Efficientnet: Rethinking model scaling for convolutional neural networks. Proceedings of the International Conference on Machine Learning.

[25] Lin T.-Y., Goyal P., Girshick R., He K., Dollar P. Focal loss for dense object detection. Proceedings of the IEEE International Conference on Computer Vision.

[26] Wu D., Lv S., Jiang M., Song H. (2020). Using channel pruning-based YOLO v4 deep learning algorithm for the real-time and accurate detection of apple flowers in natural environments. Comput. Electron. Agric..

[27] Zhang D.-Y., Luo H.-S., Cheng T., Li W.-F., Zhou X.-G., Gu C.-Y., Diao Z. (2023). Enhancing wheat Fusarium head blight detection using rotation Yolo wheat detection network and simple spatial attention network. Comput. Electron. Agric..

[28] Wang S., Zhao J., Cai Y., Li Y., Qi X., Qiu X., Yao X., Tian Y., Zhu Y., Cao W. (2024). A method for small-sized wheat seedlings detection: From annotation mode to model construction. Plant Methods.

[29] Dandrifosse S., Ennadifi E., Carlier A., Gosselin B., Dumont B., Mercatoris B. (2022). Deep learning for wheat ear segmentation and ear density measurement: From heading to maturity. Comput. Electron. Agric..

[30] Yang B., Gao Z., Gao Y., Zhu Y. (2021). Rapid detection and counting of wheat ears in the field using YOLOv4 with attention module. Agronomy.

[31] Xu X., Qiao H., Ma X., Yin G., Wang Y., Zhao J., Li H. (2023). An automatic wheat ear counting model based on the minimum area intersection ratio algorithm and transfer learning. Measurement.

[32] Gao Y. (2019). Study on Detection Method of Wheat Ear in Field Based on Deep Neural Network. Ph.D. Thesis.

[33] Maity M., Banerjee S., Chaudhuri S.S. Faster r-cnn and yolo based vehicle detection: A survey. Proceedings of the 2021 5th International Conference on Computing Methodologies and Communication (ICCMC).

[34] Zhao L., Qing S., Bai J., Hao H., Li H., Shi Y., Xing X., Yang R. (2023). A hybrid optimized model for predicting evapotranspiration in early and late rice based on a categorical regression tree combination of key influencing factors. Comput. Electron. Agric..

[35] Elfatimi E., Eryiğit R., Shehu H.A. (2024). Impact of datasets on the effectiveness of MobileNet for beans leaf disease detection. Neural Comput. Appl..

[36] Nayak A., Chakraborty S., Swain D.K. (2023). Application of smartphone-image processing and transfer learning for rice disease and nutrient deficiency detection. Smart Agric. Technol..

[37] Jin X., Bagavathiannan M., Maity A., Chen Y., Yu J. (2022). Deep learning for detecting herbicide weed control spectrum in turfgrass. Plant Methods.

[38] Wang Y., Xu X., Wang Z., Li R., Hua Z., Song H. (2023). ShuffleNet-Triplet: A lightweight RE-identification network for dairy cows in natural scenes. Comput. Electron. Agric..

[39] Hu J., Shen L., Sun G. Squeeze-and-excitation networks. Proceedings of the IEEE Conference on Computer Vision and Pattern Recognition.

[40] Yang B., Chen R., Gao Z., Zhi H. (2024). FIDMT-GhostNet: A lightweight density estimation model for wheat ear counting. Front. Plant Sci..

[41] Zhu M., Min W., Han J., Han Q., Cui S. (2024). Improved channel attention methods via hierarchical pooling and reducing information loss. Pattern Recognit..

[42] Suo J., Zhan J., Zhou G., Chen A., Hu Y., Huang W., Cai W., Hu Y., Li L. (2022). Casm-amfmnet: A network based on coordinate attention shuffle mechanism and asymmetric multi-scale fusion module for classification of grape leaf diseases. Front. Plant Sci..

[43] Verma S., Chug A., Singh A.P., Singh D. (2023). PDS-MCNet: A hybrid framework using MobileNetV2 with SiLU6 activation function and capsule networks for disease severity estimation in plants. Neural Comput. Appl..

[44] Qi Z., Hua W., Zhang Z., Deng X., Yuan T., Zhang W. (2024). A novel method for tomato stem diameter measurement based on improved YOLOv8-seg and RGB-D data. Comput. Electron. Agric..

[45] Sun X. (2024). Enhanced tomato detection in greenhouse environments: A lightweight model based on S-YOLO with high accuracy. Front. Plant Sci..

[46] Sun X., Li Y., Li G., Jin S., Zhao W., Liang Z., Zhang W. (2023). SCGNet: Efficient sparsely connected group convolution network for wheat grains classification. Front. Plant Sci..

[47] Karthik R., Vardhan G.V., Khaitan S., Harisankar R., Menaka R., Lingaswamy S., Won D. (2024). A dual-track feature fusion model utilizing Group Shuffle Residual DeformNet and swin transformer for the classification of grape leaf diseases. Sci. Rep..

[48] Yang X., Zhao W., Wang Y., Yan W.Q., Li Y. (2024). Lightweight and efficient deep learning models for fruit detection in orchards. Sci. Rep..

[49] Asker M.E., Güngör M. (2024). A hybrid approach consisting of 3D depthwise separable convolution and depthwise squeeze-and-excitation network for hyperspectral image classification. Earth Sci. Inform..

[50] David E., Madec S., Sadeghi-Tehran P., Aasen H., Zheng B., Liu S., Kirchgessner N., Ishikawa G., Nagasawa K., Badhon M.A. (2020). Global wheat head detection (GWHD) dataset: A large and diverse dataset of high-resolution RGB-labelled images to develop and benchmark wheat head detection methods. Plant Phenomics.

[51] David E., Serouart M., Smith D., Madec S., Velumani K., Liu S., Wang X., Espinosa F.P., Shafiee S., Tahir I.S. (2021). Global wheat head dataset 2021: More diversity to improve the benchmarking of wheat head localization methods. arXiv.

[52] Wenxia B., Wenjie X., Gensheng H., Xianjun Y., Biaobiao S. (2023). Wheat ear counting method in UAV images based on TPH-YOLO. Trans. Chin. Soc. Agric. Eng..

[53] Li J., Dai F., Qian H., Huang L., Zhao J. (2024). Lightweight Wheat Spike Detection Method Based on Activation and Loss Function Enhancements for YOLOv5s. Agronomy.

[54] Jie L., Zihao Y., Quan Z., Jiangwei Q., Jingmin T. (2024). Method for detecting and counting wheat ears using RT-WEDT. Trans. Chin. Soc. Agric. Eng..

[55] Wen C., Ma Z., Ren J., Zhang T., Zhang L., Chen H., Su H., Yang C., Chen H., Guo W. (2024). A generalized model for accurate wheat spike detection and counting in complex scenarios. Sci. Rep..

[56] Zhao J., Ren R., Wu Y., Zhang Q., Xu W., Wang D., Fan L. (2024). SEAttention-residual based channel estimation for mmWave massive MIMO systems in IoV scenarios. Digit. Commun. Netw..

[57] Das D., Nayak D.R., Bhandary S.V., Acharya U.R. (2024). CDAM-Net: Channel shuffle dual attention based multi-scale CNN for efficient glaucoma detection using fundus images. Eng. Appl. Artif. Intell..

[58] Zhang J., Li X., Liu D., Yu S. Road Target Detection Algorithm Based on Improved YOLOv5. Proceedings of the 2023 IEEE International Conference on Unmanned Systems (ICUS).

